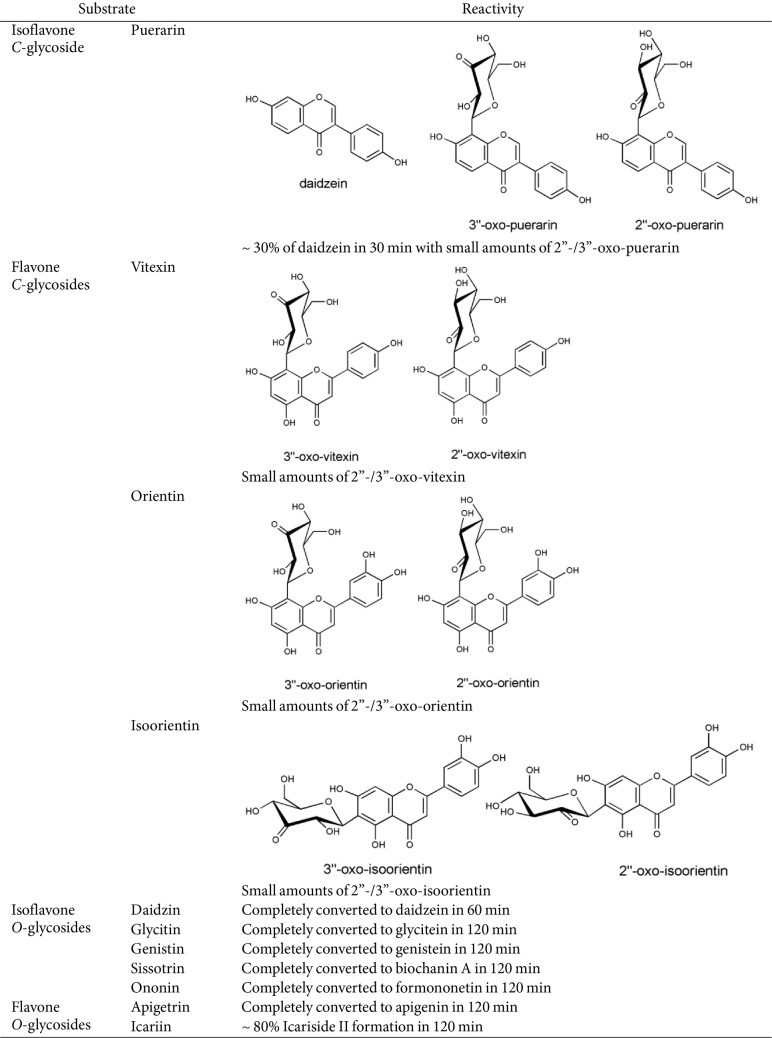# Flavonoids Biotransformation by Human Gut Bacterium Dorea sp. MRG-IFC3 Cell-Free Extract

**DOI:** 10.4014/jmb.2403.03058

**Published:** 2024-04-27

**Authors:** Huynh Thi Ngoc Mi, Heji Kim, Jong Suk Lee, Bekir Engin Eser, Jaehong Han

**Affiliations:** 1 Metalloenzyme Research Group and Department of Plant Science and Technology, Chung-Ang University, Anseong 17546, Republic of Korea; 2 Bio Industry Department, Gyeonggido Business & Science Accelerator (GBSA), Suwon 16229, Republic of Korea; 3 Department of Biological and Chemical Engineering, Aarhus University, 8000 Aarhus C, Denmark

**Keywords:** C-C bond cleavage, *Dorea* sp. MRG-IFC3, flavonoid glycoside, puerarin

## Abstract

Human gut bacterium *Dorea* sp. MRG-IFC3 is unique in that it is capable of metabolizing puerarin, an isoflavone *C*-glycoside, whereas it shows broad substrate glycosidase activity for the various flavonoid *O*-glycosides. To address the question on the substrate specificity, as well as biochemical characteristics, cell-free biotransformation of flavonoid glycosides was performed under various conditions. The results showed that there are two different enzyme systems responsible for the metabolism of flavonoid *C*-glycosides and *O*-glycosides in the MRG-IFC3 strain. The system responsible for the conversion of puerarin was inducible and comprised of two enzymes. One enzyme oxidizes puerarin to 3”-oxo-puerarin and the other enzyme converts 3”-oxo-puearin to daidzein. The second enzyme was only active toward 3”-oxo-puerarin. The activity of puerarin conversion to daidzein was enhanced in the presence of Mn^2+^ and NAD^+^. It was concluded that the puerarin *C*-deglycosylation by *Dorea* sp. MRG-IFC3 possibly adopts the same biochemical mechanism as the strain PUE, a species of *Dorea longicatena*.

## Introduction

Gut metabolism of bioactive natural products has provided a new opportunity, leading to the discovery of novel chemical conversions and new bioactive compounds [1-3]. Likewise, *C*-glycosidic bond cleavage by gut bacteria has been a challenge for chemists, because it is unprecedented in the realm of chemical reactions. Since the first report of aloin *C*-deglycosylation [4], quite a few gut bacteria have been reported to convert various natural *C*-glycosides to the aglycones [5]. Recently, a series of scientific reports on puerarin conversion to daidzein has elucidated splendidly that the cleavage of *C*-glycosidic bond occurs not by hydrolysis, but by E1cB elimination reaction of 3”-oxo-puerarin [6-8]. Namely, no direct deglycosylation of puerarin occurs and the oxidation to 3”-oxo-puerarin appears to be requisite for the biochemical puerarin metabolism (Scheme 1).

Our continuing investigation of the gut metabolism of dietary natural products has resulted in the elucidation of biosynthetic pathway of S-equol, for the first time [3], and the discovery of O-demethylation of methylated phenylpropanoids [9, 10]. In addition, we have reported *C*-deglycosylation of puerarin by gut bacteria [11]. Recently it was found that *Dorea* sp. MRG-IFC3 is similar to the PUE strain by 85% of rDNA sequence homology, but its reactivity of *C*-glycoside conversion was different in terms of substrate specificity [12]. While the *C*-deglycosylation by other human gut bacteria was reported to exhibit a broad substrate spectrum for various *C*-glycosides, *Dorea* sp. MRG-IFC3 has metabolized only puerarin.

Even though *Dorea* sp. MRG-IFC3 has metabolized various flavonoid *O*-glycosides, similar to the reported gut bacteria which can metabolize *C*-glycosides, high substrate specificity for *C*-glycoside conversion encouraged us to investigate biochemical characteristics of this enzymology. Especially, the substrate specific transporter in the cell membrane was hypothesized to explain the specificity of *C*-deglycosylation by *Dorea* sp. MRG-IFC3. Therefore, cell-free extract biotransformation was performed to test the hypothesis and to provide basic biochemical characteristics of the *C*-glycosidic bond cleavage reaction.


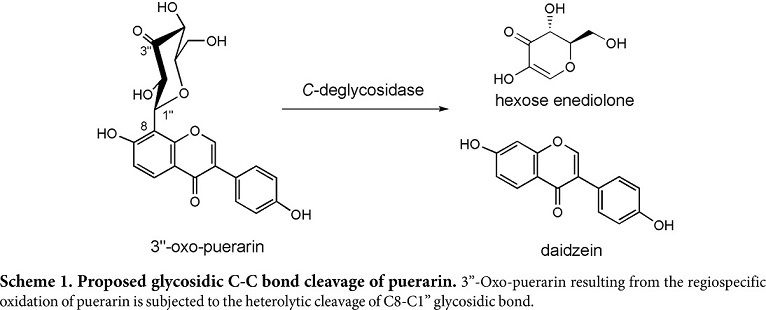


## Materials and Methods

### Chemicals

Extra pure grade ethyl acetate (EtOAc; >99.80%), and methanol (MeOH; 99.50%) were purchased from Daejung Co., Ltd. (Republic of Korea). HPLC grade *N, N*-dimethylformamide (DMF) were purchased from Samchun Pure Chemical Co., Ltd. (Republic of Korea). HPLC grade acetonitrile (MeCN), methyl alcohol (MeOH), and water were purchased from Burdick & Jackson (USA). Acetic acid for HPLC was purchased from Sigma-Aldrich (Switzerland), while formic acid for mass spectrometry (~98%) was purchased from Fluka (Germany). Puerarin (daidzein-8-*C*-glucoside) and daidzin (daidzein-7-*O*-glucoside) were from Sejin CI (Republic of Korea). Apigetrin (apigenin-7-*O*-glucoside), glycitin (glycitein 7-*O*-glucoside), genistin (genistein 7-*O*-glucoside), ononin (formononetin 7-*O*-glucoside), and sissotrin (biochanin A 7-*O*-glucoside) were purchased from Indofine Chemical Co. (USA). Vitexin (apigenin-8-*C*-glucoside) was obtained from Fluka (Sigma-Aldrich Co., USA). Gifu anaerobic medium (GAM) from Nissui Pharmaceutical Co. (Japan) was used for isolation and as growth media. GAM broth was prepared according to the manufacturer’s instructions, and the GAM plate contained 15 g l^−1^ agar in GAM broth. For the preparation of GAM plate, 1.5% (w/v) of agar (Junei Chemical, Japan) was added to GAM broth medium. All other chemicals were of analytical reagent grade.

### Activity of Cell-Free Extract

All the experimental procedures, including screening, isolation, and identification of bacteria, were performed under anaerobic conditions (CO_2_ 5%, H_2_ 10%, N_2_ 85%) at 37°C, except HPLC analysis. For the cell-free extract, *Dorea* sp. MRG-IFC3 was cultured under anaerobic conditions at 37°C for 20 h in 1 L of GAM broth containing 128.8 mg (0.3 mM) puerarin as an enzyme inducer. The bacteria cells were collected by centrifugation (7000 ×*g*, 10 min) when the OD_600_ reached 2.2. The harvested pellet was washed with 50 mM phosphate buffer (pH 7.4, 50 ml) before storage in liquid nitrogen. For the cell lysis, cell pellet (2 g) was resuspended in 10 ml of phosphate buffer (50 mM, pH 7.4), and disrupted by sonication at 60% amplitude for 30 min with a cycle of 10 sec on and 20 sec off at 0°C. The cell lysate was then centrifuged at 13,000 g for 90 min at 4°C to obtain the supernatant as a cell-free extract. The cell-free extract was also prepared under argon atmosphere to test the air-sensitivity. In detail, degassed buffers were prepared by vacuum evacuation with sonication, and centrifugation was performed under argon. The reaction was performed in the anaerobic chamber. The cells without puerarin inducer were processed by the same method, except that the culture broth did not contain puerarin.

To check the activity of cell-free extract, 0.2 mM of each substrate was reacted at 37°C. Aliquots of the reaction (100 μl) were taken, allocated into microcentrifuge tubes, and extracted with 1 ml ethyl acetate. Then, 800 μl supernatant, collected after vortexing and centrifuging at 10,770 g for 10 min, was dried under vacuum. The dried residue was dissolved in 100 μl MeOH, and filtered through a 0.2 μm PTFE syringe filter (Advantec, Japan) for the analysis by HPLC.

HPLC analysis was conducted by UHPLC-DAD (Thermo Fisher Scientific, USA), with a kinetex C18 column (1.7 mm particle size; 100 × 2.1 mm i.d., USA) at 35°C. The flow rate was 0.2 ml/min with the mobile phase consisting of 0.1% formic acid (v/v) in water (A) and acetonitrile (B). The eluent started with solvent B from 5 to 55% in 20 min and was held at 55% for 5 min, then increased linearly to 100% in 5 min and maintained for 3 min. After the analysis, the composition of the eluent was returned to 5% B in 2 min linearly. The injection volume was 1.0 ml. Program setup, data collection, and analysis were performed using Chromelon Chromatography Data System software version 6.8 (Thermo Fisher Scientific).

### Cell-Free Extract Reaction in the Presence of Additives

Cell-free extract was incubated with 0.2 mM of each substrate (puerarin or daidzin) in 50 mM potassium phosphate buffer (pH 7.4) containing 1 mM Mn^2+^ and 1 mM NAD^+^ at 37ºC. Aliquots of the reaction mixture (100 μl) were taken, allocated into microcentrifuge tubes, and extracted with 1 ml ethyl acetate. Then, 800 μl supernatant was collected after vortexing and centrifuging at 10,770 g for 10 min, followed by drying under vacuum. The dried residue was dissolved in 100 μl MeOH filtered through a 0.2 μm PTFE filter (Advantec, Japan) for the chromatography analysis by HPLC.

## Results and Discussion

Currently, three gene clusters, *dfg*, *dgp*, and *car* from *Eubacterium cellulosolvens*, *Dorea* strain PUE and *Microbacterium* strain 5-2b, respectively, are reported to exhibit *C*-deglycosylation activity [6, 13, 14]. However, DgpA and DgpBC from *dgp* operon are the only enzymes of which the biochemical properties were investigated at the molecular level. Thus, our understanding of biochemical *C*-deglycosylation is limited to the mechanism proposed from the strain PUE, so that it is not certain whether other natural *C*-glycosides would follow the same biochemical mechanism. In this report, the results obtained from flavonoid biotransformation by the cell-free extract of *Dorea* sp. MRG-IFC3 have provided a number of significant findings.

### Puerarin *C*-Deglycosidase from *Dorea* sp. MRG-IFC3 is Inducible

**Fig. 1:**
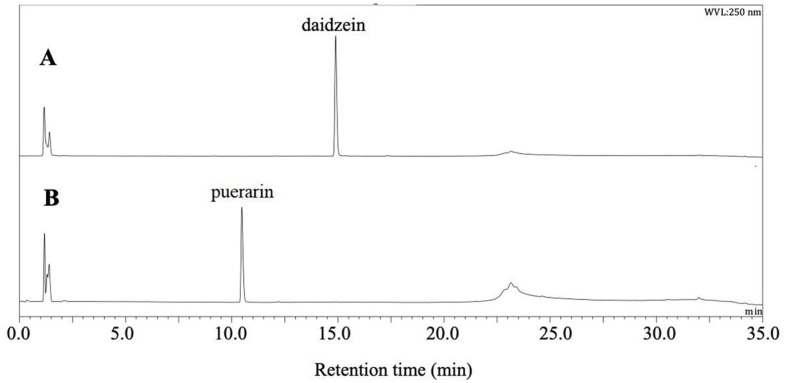
Puerarin biotransformation by cell-free extracts of *Dorea* sp. MRG-IFC grown in the presence (**A**) and in the absence (**B**) of puerarin. The reaction time was 60 min.

*Dorea* sp. MRG-IFC3 is a strict anaerobe. First, air-sensitivity of *C*-deglycosylation was tested by comparing the reactivity of cell-free extracts in the presence and absence of air. The activity of conversion of *C*/*O*-glycosides, such as puerarin and daidzin, was not affected by air. Therefore, it was determined that glycoside metabolizing activity does not require the air-sensitive cofactors, such as Fe/S cluster. Secondly, inducibility of *C*-deglycosylation was investigated by comparing the puerarin conversion activity of the cell-free extracts prepared from the cells grown in the presence and absence of puerarin. The cell-free extract prepared from the cells grown with 0.3 mM puerarin completely converted puerarin in 60 min (Fig. 1A), whereas that from the cell grown without puerarin did not show any activity at all (Fig. 1B). Therefore, it was concluded that the enzyme responsible for puerarin conversion to daidzein is inducible.

Furthermore, it was found that the enzyme responsible for *O*-glycoside conversion is different from the *C*-deglycosidase. Both cell-free extracts, prepared from the cells grown in the presence and absence of puerarin, exhibited the same *O*-glycoside conversion activity. When time-dependent *C/O*-glycoside conversions by the cell-free extract were compared, the conversion of *C*-glycosides puerarin was slower than that of *O*-glycoside daidzin (Table 1). Besides, puerarin conversion was not completed even after depletion of daidzin. The results confirmed that the activities of *C/O*-glycosidic bond cleavage by *Dorea* sp. MRG-IFC3 are achieved by different enzymes and the enzyme responsible for the *C*-glycoside metabolism is inducible.

### Puerarin *C*-deglycosidase from *Dorea* sp. MRG-IFC3 only Converts Isoflavone C-Glycoside Puerarin

**Fig. 2:**
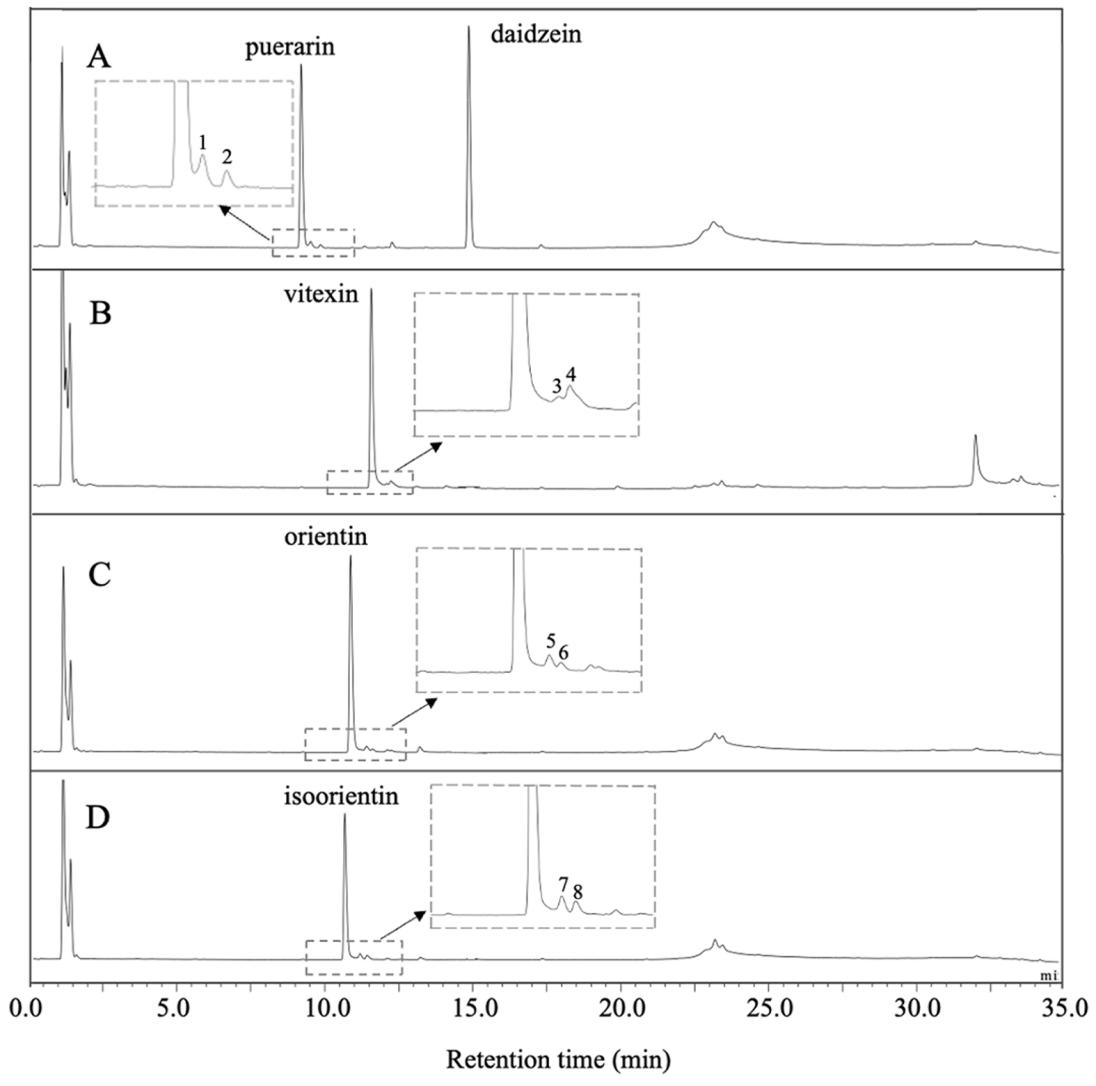
HPLC chromatograms of C-glycosides reaction products of the induced cell-free extract. After 2 h of reaction time, puerarin (**A**) was converted to two minor products (peak 1 and 2) as well as daidzein. Vitexin (**B**) was converted to two minor products (peak 3 and 4), orientin (**C**) was converted to two minor products (peak 5 and 6), and isoorientin (**D**) was converted to two minor products (peak 7 and 8).

As reported previously from whole cell biotransformation, *Dorea* sp. MRG-IFC3 metabolized only puerarin, an isoflavone *C*-glycoside. It does not metabolize flavone *C*-glycosides, such as vitexin, orientin and isoorientin [12]. One of the possible explanations was the cell transporter which is specific to puerarin, similar to the cell signaling during the nodule formation of the symbiotic nitrogen-fixing soil bacteria [15]. When we measured the reactivity of cell-free extract with various *C*-glycosides, no difference has been observed, except reaction rate of puerarin conversion (Fig. 2). Therefore, it was concluded that the high substrate specificity by *Dorea* sp. MRG-IFC3 *C*-deglycosidase toward C-glycosides is an intrinsic property.

On the other hand, all the *O*-glycosides, including flavone *O*-glycosides and isoflavone *O*-glycosides, were converted by the cell-free extract and the aglycones were produced. No other possible peaks for byproducts or intermediates were observed from the reaction of *O*-glycosides (Table 1).

### Puerarin *C*-deglycosidase from *Dorea* sp. MRG-IFC3 May Adopt the Same Mechanism as *C*-Deglycosidase of the Strain PUE

Previously, no intermediate was observed during the whole cell biotransformation of *C*-glycosides [12]. Interestingly, two minor metabolites were observed from the cell-free extract biotransformation of *C*-glycosides (Fig. 2). These two metabolites, detected right after the substrates on the HPLC chromatograms, were identified as 3”- and 2”-oxo-products by LC-MS analysis, as reported by others [6, 13, 16]. Except puerarin (Fig. 2A), no aglycone productions for the other three *C*-glycosides were observed (Fig. 2B-2D). On the contrary, the oxo-products were not observed from *O*-glycosides reactions by cell-free extract, which again confirmed that *C*-/*O*-glycosides metabolism by *Dorea* sp. MRG-IFC3 are performed by different enzyme systems.

**Fig. 3:**
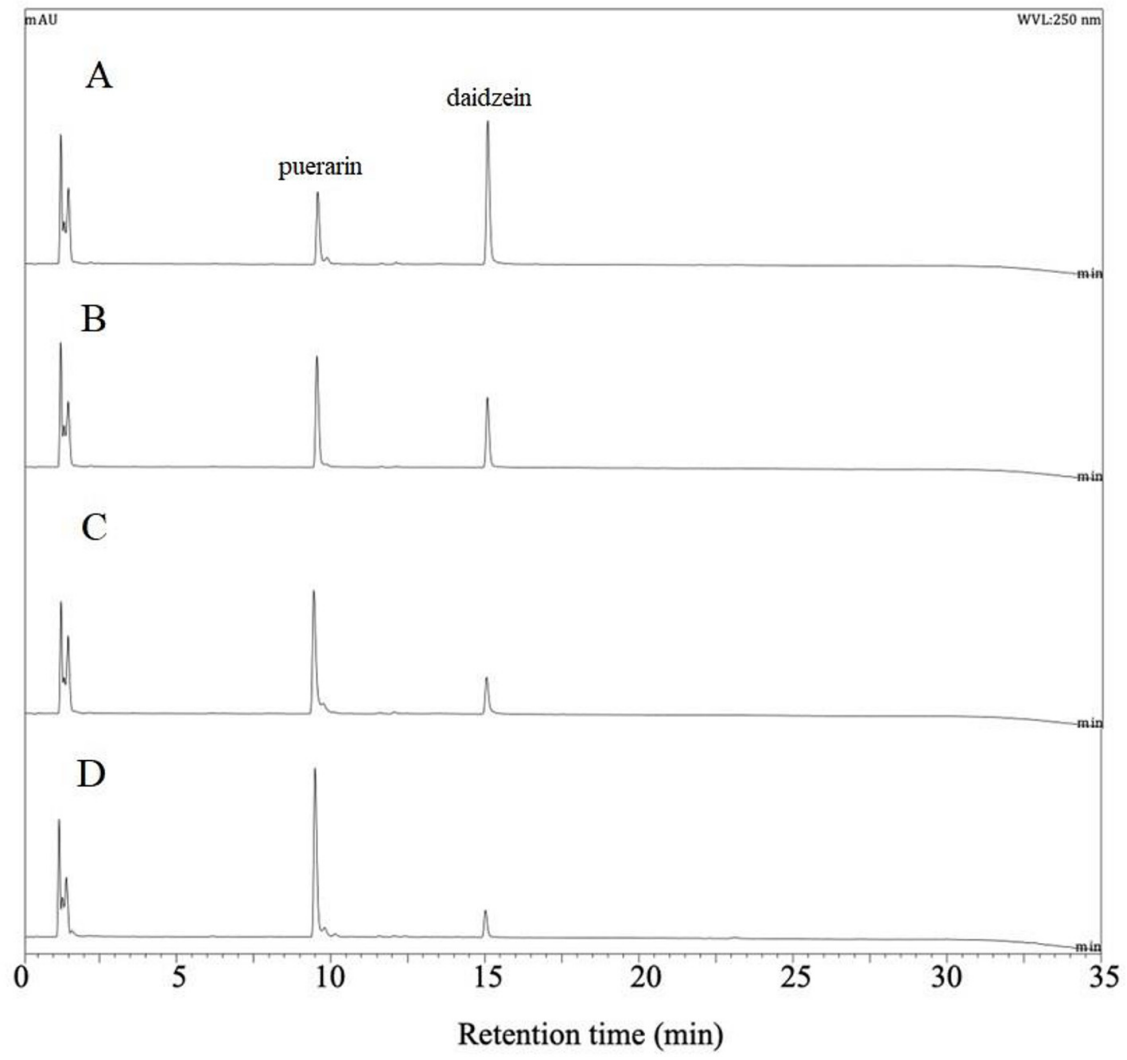
HPLC chromatograms of puerarin reaction products by the induced cell-free extracts of *Dorea* sp. MRG-IFC3 in the presence of NAD^+^ and/or Mn^2+^. Daidzin production from puerarin was highest in the presence of NAD^+^ and Mn^2+^ (**A**) compared to the reactions with only Mn^2+^ (**B**) or only NAD^+^ (**C**). The reaction without any cofactors (**D**) was performed as control experiment.

The activity of puerarin biotransformation by the cell-free extract was influenced by the additives, Mn^2+^ and NAD^+^ and both significantly increased the daidzein production (Fig. 3). NAD^+^ was proposed as a cofactor of DgpA glycoside oxidoreductase and Mn^2+^ as a cofactor of DgpBC *C*-deglycosidase from the strain PUE [7, 8]. Besides, it is noteworthy that the amounts of 3”-oxo-puerarin was significantly reduced in the presence of Mn^2+^ (Fig. 3B). Therefore, it is proposed that *C*-deglycosylation of *Dorea* sp. MRG-IFC3 follows the same reaction mechanism as the strain PUE. Though, it is still not clear why it does not react with other *C*-glycosides. Based on our observation shown at Fig. 2, it is clear that the other *C*-glycosides, such as vitexin, orientin, and isoorientin, were converted to the oxo-intermediates. Hence, we propose that the substrate binding site of *C*-deglycosidase of *Dorea* sp. MRG-IFC3 is different from that of the PUE strain [7].

## Conclusion

Biochemical *C*-deglycosylation is known to occur in two steps [7]. Namely, puerarin needs to be oxidized to 3”-oxo-puerarin, before the cleavage reaction of the glycosidic C-C bond. The former reaction was proposed being catalyzed by NAD^+^-dependent oxidoreductase DgpA, and the latter performed by Mn^2+^ ion-dependent *C*-deglycosidase DgpBC in the strain PUE. Because *Dorea* sp. MRG-IFC3 strain belongs to the same family of PUE, the substrate specificity of *C*-glycoside metabolism has been investigated in this report, by means of cell-free extract biotransformation.

As shown by the results, it was concluded that two different enzyme systems are responsible for the metabolism of flavonoid *C*-glycosides and *O*-glycosides in *Dorea* sp. MRG-IFC3. The system for the puerarin conversion was inducible and appeared to be comprised of two enzymes, the one oxidizing puerarin to 3”-oxo-puerarin and the other converting 3”-oxo-puearin to daidzein. Based on the cofactor requirement and the production of 3”-oxo-*C*-glycoside intermediate, puerarin *C*-deglycosylation by *Dorea* sp. MRG-IFC3 was expected to follow the same biochemical mechanism as the strain PUE. Besides, it was proposed the enzyme responsible for the cleavage of the *C*-glycosidic bond is active only to 3”-oxo-puerarin, resulting in the high substrate specificity of *C*-deglycosylation by *Dorea* sp. MRG-IFC3.

## Figures and Tables

**Table 1:** Reactivity of *Dorea* sp. MRG-IFC3 cell-free extract prepared from the induced cells.